# Observations on the Surgical Anatomy of the Head and Neck

**Published:** 1816-03

**Authors:** 


### *Observations on the Surgical Anatomy of the Head and Neck.* By Mr. Allan Burns.

( Continued from page 68 )

In conformity with the mode of examination proposed and adopted in our Number for January, we proceed to notice the pathological and therapeutic portion of this interesting volume. In our former article was traced the surgical anatomy,—the present will comprehend the surgical pathology, of the cervical and facial regions. For the sake of order and perspicuity, we shall assign to each subject a distinct section; and concentrate under it all the light which the talents of Mr. Burns may have thrown upon such deparment of his investigations. It is much to be regretted, that he did not adopt a plan thus calculated to enhance the value of his labors.

*Pathology of the cervical fascia.*—The principal office of the sterno-hyoid and thyroid muscles, is to co-operate with the fascia in counteracting the pressure of the external air on the trachea. While these are entire, respiration, under ordinary circumstances, is undisturbed. If they are destroyed, the unresisting integuments are pressed inwards by the atmosphere, on every effort to inspire; and serious dyspnœa results from compression of the tracheal tube. These notions are illustrated by a case wherein destruction of the fascia and muscles was the consequence of abscess of the anterior mediastinum bursting above the sternum. The lost substance was not restored; and, after the wound had closed, merely a thin pellicle of polished skin covered the trachea, arteria innominata, and thyroid branch of the inferior thyroid artery. Hence difficult respiration arose, or rather was aggravated: for the subject of this observation had suffered dyspnœa from the period of an attack of chincough about his third year. The irritation of this disease had probably excited chronic enlargement and suppuration of the thymus gland. Mr. Burns thought, that the loss of the fascia and muscles in such a case might be supplied by a piece of leather spread with sticking-plaster. This should be adjusted to the part during *expiration;* retained *in situ* till firmly fixed; and the insinuation of air beneath its edges prevented by a brushing over with the alcoholic solution of sealing-wax. The experiment had never been made.

Tumors about the throat may be situated exteriorly to the fascia; behind it; or between the two plates of which it consists inferiorly. Like tumors in other parts, they may be simple or specific, primary or secondary. In the present state of our knowledge respecting morbid structure, no criteria are ascertained which can fix the diagnosis between simple and specific tumors. The classification of Mr. Abernethy is objectionable, inasmuch as actual examination of a tumor is necessary to determine its nature; and those may present externally characters precisely similar, which differ essentially in their internal structure. This difficulty is much to be lamented: for, while the surgeon may succeed in removing the simple tumor by the processes of absorption or suppuration, the only prospect of treating with success the specific, is extirpation, ere from size, connections, or noxious influence on the surrounding parts, it has acquired a formidable aspect. Procrastination, under such circumstances, is dangerous; often fatal. Hence, every clue that can assist us in deciding upon this important question, should be seized. Nor should the nature of secondary tumors be less sedulously explored. But here the field of inquiry is more limited; the sources of doubt less numerous. If the primary disease be simple, so must the secondary: yet, where the primary is specific, the secondary is not invariably the same: it may result either from absorption or sympathetic irritation. In the former case, the secondary swelling will partake the malignant character of its predecessor, and must be removed by an operation: in the latter, it will be simple, and yield to gentler means. Mr. Burns thought, "that the lymphatic glands never become *specifically* contaminated previous to the formation of an *ulcer* or *fungus* in the part primarily affected;" although they may swell at an early period, from sympathy:—that in fact, there is specific absorption, but no specific irritation Hence, if the primary tumor be not ulcerated externally; and if after removal, minute inspection detect no internal fungi or ulcerations, it will be proper to defer the excision of any secondary swelling that may exist, in the hope of its being merely the effect of sympathetic irritation, and thus requiring for its removal only the ordinary treatment of simple tumors. Diagnosis of tumors about the neck is, however, exceedingly difficult; and, on account of the vicinity of large vessels and nerves, they demand especial vigilance. In all dubious cases, it is most prudent to aim at procuring absorption; but, if this attempt fail, time should not be lost in efforts to induce suppuration, as the efficacy of this process is restricted to simple tumors, or, in other words, to those only which require extirpation from their mechanical influence on some neighbouring organ. Danger is generally past when in a simple swelling suppuration can be established.

Tumors which arise from preternatural deposition of substance resembling the original texture of the part, are commonly indolent, and seldom assume an active character unless improperly irritated: Those, which consist of morbid structure, are naturally disposed to inflame and pass on to the sphacelating, fungous, or ulcerative state: Upon this difference of character an useful distinction of tumors might be founded, were we able from an external view to determine the peculiarity of internal organization.

These peculiarities as to the structural and morbid phænomena, diagnosis, and treatment, of tumors having been reviewed, we are next to consider their variety of situation with respect to the cervical fascia, and the influence which that membrane exerts in modifying, according to such situation, their external developement and features, and in regulating the views of the surgeon in his removal of them.

*Tumors situated exteriorly to the fascia,* are superficial, mobile; spread laterally as they grow; are circumscribed, elevated, capable of being, for a long time, grasped and drawn out; and hence readily extirpated. They should be removed early, if of a malignant nature; for by pressing on the fascia in their increase, they form adhesion to it; sometimes get behind it by absorption of the interposed membrane, and become deeply connected as though originally placed there: yet sometimes even with large tumors no adhesions take place. These tumors may be readily removed from any part of the throat, while unadhering. They commonly arise from enlargement of a subcutaneous gland; are firm and unyielding from the small quantity of interstitial fluid, or only slightly elastic; indolent, painless, not prone to suppurate, sometimes exciting inflammation in contiguous parts; and thus, deprived of the supply of blood, spontaneously thrown off. When not destroyed, they progressively increase; and from a new mode of vascular action, assume a cartilaginous or bony structure. They consist internally of a mixture of cellular and intervertebral substance; have few nerves and arteries; the latter very small and not requiring ligature; but the veins often varicose and causing the only inconvenience attendant on their extirpation. In this operation, too much skin should not be left: hence, if the tumor be large, a portion of integument, even though healthy, should be removed; and, in dividing its cellular connections with the fascia, the left hand should be employed in pulling it outwards. Where the tumor is small, it may, after proper exposure by dissection, be advantageously grasped and wrenched by a twisting motion from its connections. Thus the pain will sooner cease; and hæmorrhage be prevented. Sticking plaister, not sutures, is to be used in keeping the lips of the wound closed; and a compress and bandage will preserve the integuments in contact with the parts beneath.

*Tumors originating between the layers of the fascia* from enlargement of the conglobate glands imbedded there, may be mistaken for a diseased thymus gland: but enlargement of the thymus to such a degree, would induce serious dyspnœa; and the lymphatic tumor, as it will be commonly easy to ascertain, has no connection with the chest. External to the deep fascia and muscles, and implicating no great nerve or vessel, such a tumor may without danger be started from its capsule.

*Tumors beneath the fascia* frequently occur. Restricted and flattened externally by that membrane, they do not protrude until they have acquired a large size; and are deeply connected beneath. Hence, while their apparent size is trifling, they often stretch backward far and wide amid muscles, nerves, and vessels; and produce dysphagia and disordered respiration by pressure upon the œsophagus and trachea. Hence too, if such a tumor be specific, and on the increase, the propriety of removal cannot be doubted, other circumstances being favorable to the operation. Even simple tumors, seated here, may require excision, if they resist ordinary treatment, and become so large as to compress the important parts beneath. But in an advanced state of the disease such an operation is dangerous and perplexing; in some cases absolutely desperate. The necessity of speedy removal of specific swellings, therefore, cannot be too strongly urged; for, should the morbid action have extended to remote parts, the complete removal of the disease can never be with certainty accomplished; "and if a single atom of the contaminated parts be permitted to remain, the patient is in a condition equally dangerous as before we operated. The disease is suspended, not eradicated, and the secondary affection is worse than the first." Subfascial tumors arising in the triangular space which, in our last Article, [Page 54] we noted as being divided by the posterior belly of the omo-hyoid muscle, are peculiarly difficult of extirpation. Bound down by that muscle and the fascia, a swelling thus circumstanced does not soon project externally; but, at length presenting, pushes before it the omo-hyoid muscle, turns forward the acromial border of the sterno-mastoid, and pulls away the clavicular from the sternal portion.

"Wherever, therefore, the sterno-mastoid is seen pulled over a tumor situated between that muscle and the trapezius, the surgeon may be certain that it is deeper seated than the omo-hyoideus."

The attachments of such a tumor it is difficult to determine precisely. If, however, on grasping it with one hand and moving it from side to side, the fingers of the other, introduced deep behind the clavicle, find the subclavian artery unaffected by the motions of the tumor, and consequently beating always in one spot, no adhesion, it may be inferred, has taken place between the latter and the vessel: and, if the arm be free from numbness, the tumor is probably unconnected with the subclavian plexus of nerves. But where the tumor originally sprang up just above the clavicle, or has become so large as to be "jammed in" behind that bone, its connections cannot be made out; and a glandular swelling here, as it receives the impulse of the subclavian artery, may be mistaken for aneurism. Of this error an instance is recited by Mr. Burns. In removing such tumors, the knife should only be used in fairly exposing them. The detachment of the morbid substance is then to be effected by the fingers: it should be torn "from the tracheal towards the acromial side," so as to follow the course of the vessels and nerves: and, after removal, the surgeon should cautiously ascertain that no portion of it, no enlarged gland, or indurated cellular membrane, has been left behind; but he must not mistake "the ruptured arteries which are felt like small hard points projecting from the wound, for specks of diseased matter."*

The conglobate glands, situated between the sterno-thyroid muscle and œsophagus, sometimes enlarge, and produce serious dysphagia. Such a tumor is deep-seated; and has, occasionally, an appearance of diffusion greater than really exists. It frequently suppurates; and the abscess, in which fluctuation is almost imperceptible, commonly bursts into some deeply-seated part, as the gullet or wind-pipe: the matter has even been poured into the jugular vein. In one case, where the abscess had burst into the trachea, the patient "could inflate the sac; he was teazed with cough, expectorated purulent matter, and died hectic." Where, therefore, resolution cannot be accomplished, and an abscess forms, the surgeon must not delay till fluctuation becomes distinct; but discharge the matter as early as possible. The puncture must be cautiously made; for the thyroid branch of the inferior thyroid artery has been seen pushed out before the cyst of such an abscess.

Enlargement of the *glandulæ concatenatæ* accompanying the carotid artery may be mistaken for aneurism. The symptoms, in a case of this kind, noted by Mr. Burns, were slight pain and fulness on the left side of the thyroid cartilage, succeeded by a swelling, which pulsated strongly. Several believed it to be aneurism: but accurate examination shewed that it was "alternately ele-

---

* A curious and fatal affection of one of the glands situated externally to the fascia over this triangular space, is recorded by Mr. Burns, page 51. It was complicated, towards the close, with general emphysema.

vated and depressed by the action of the carotid," and that " the rising and falling did not depend on any variation in the magnitude of the swelling itself." Lateral pressure did not diminish its volume ; and by pulling it forward from the sphere of arterial action, all trace of pulsation was lost. Thus it was distinguishable from an aneurismal tumor, which " becomes alternately tense and puffed up, and smaller and more flaccid." It is probable, that many of the supposed cases of aneurism, terminating by spontaneous cure, were, in fact, glandular swellings, situated on the course of a large artery. Where such a tumor is placed in the middle region of the neck, the pulsation will frequently be violent; but more obscure, if its site be above the line of the digastric muscle, or below the decussation of the sterno mastoid and omo-hyoid : for here the artery and glands are deep-seated. But the apparent pulsation of a tumor, even in the middle cervical region, will subside when it becomes too large to be longer affected by the oppressed action of the artery. Projection of the vessel *in front* forms the only circumstance under which such a swelling, when large, can retain its seeming pulsation; and here, from the " defined course of the pulsation," the diagnosis will be clear. Breathing and deglutition are soon affected by a tumor of the *glandulæ concatenatæ* ; and by its pressure on the eighth and sympathetic nerves, the functions of the chylo-poietic viscera are deranged.* Hence the most vigorous means should be adopted to remove it. These failing, extirpation must be resorted to without loss of time ; for it can only succeed in the earlier stages of the complaint. Connection with the artery, which may be ascertained in the way before indicated, would forbid the operation. The tumor, if affecting a gland originally *behind* the carotid artery, sometimes rises up on each side of that vessel, " so as to bury it, the jugular vein, the nervus vagus, and ramus descendens noni in the very centre of the morbid parts." All the connections of the tumor should, therefore, be clearly ascertained previously to an operation; and, if these be favourable, delay is out of the question.

" Proceed decidedly to its extirpation; place the patient in a proper position; make then an incision through the integuments,

---

* Upon this point, probably, Mr. Abernethy would not coincide with our author. By that gentleman, the disorder of the chylo-poietic functions would be regarded as the source of the tumor.

the platysma myoides, and the fascia, down to the tumor, which next expose, by dissecting back the parts which cover it. Now lay aside the knife; act with boldness and decision; grasp the tumor firmly with the finger and thumb of the right hand; ascertain that the hold is secure, and instantly and steadily wrench it from its attachments behind."

This advice is excellent, and cannot be too strongly impressed upon the recollection of surgeons. From inattention to this mode of operating, we have seen, in the removal of a small glandular swelling at the base of the lower jaw, the external maxillary and lingual arteries cut through, (the latter about one-fourth of an inch from its origin) and the structure of the submaxillary gland woefully mangled. In this case, the flow of blood from the divided lingual artery was almost as tremendous as though the trunk of the carotid had been opened; and we were preparing to cut down upon the latter vessel, when a ligature was fortunately, after many fruitless attempts, passed round the short stump of the lingual. We trust, that on the remembrance of some others, as well as on our own, the particulars of this bloody business have been deeply and usefully engraven!

The small gland, situated between the os hyoides and thyroid cartilage, and beneath the hyo-thyroid muscle,* when it becomes enlarged, is so tightly bound down by that muscle, the cervical fascia, and platysma myoides, as to produce dreadful effects on breathing and deglutition. Mr. Burns has twice seen it affected with fungus hæmatodes. One of the cases is detailed at length, and possesses uncommon interest. The patient fell a victim to the ignorance of a surgeon, on whose opinion she unfortunately relied; and who dissuaded her from submitting to an operation at the period when it might have been undertaken with an almost certain prospect of success. We exhort every surgeon to read attentively, and to digest the observations of Mr. Burns on this deplorable case. They may be found at page 91—93.

Uneasiness in swallowing, and slight pain of the throat on pressure, were here the precursory symptoms. At length, a firm, elastic, flattish tumor arose to the left of the thyroid cartilage; adhering to, and covering three-fourths of its lateral flap, and occupying the whole space between that cartilage and the hyoid bone. The finger, thrust

---

* See our last article, page 57.

deep behind the mouth, felt the tumor projecting into the pharynx, a little below the angle of the jaw, close on the arytenoid cartilage and root of the epiglottis. Pressure upon it obstructed respiration, which was always performed "with a hoarse, whizzing noise." Deglutition was uniformly difficult. The pain was lancinating and terrible. The tumor made little progress externally, but encroached farther on the pharynx, so as to destroy the miserable woman, whose last agonies are depicted by Mr. Burns in a very striking and expressive manner. An engraving presents two clear views of the morbid gland and its connections. In removing such a tumor, the surgeon would have to divide the integuments, platysma myoides, and fascia. The superior laryngeal artery and nerve are all that can be certainly injured in tearing it out.* Sometimes, in the event of strong adhesion of the tumor to the membrane extended between the hyoid bone and thyroid cartilage, the removal of that membrane might be necessary; but such an opening into the fauces is of little consequence, since the patient may be supported till it have again closed, by the elastic gum tube passed through the nostril into the gullet.

*The thymus gland* is subject to scrofulous enlargement. Repressed anteriorly by the sternum; above, by the fascia and muscles, the gland, under these circumstances, presses backwards on the organs between it and the spine; and the patient perishes from want and suffocation. In three cases, Mr. Burns has discovered a co-existent obstruction and swelling of the mesenteric glands, which he attributed to the pressure of the morbid thymus on the subclavian vein, "interrupting the entrance of the chyle into the heart." Repeated blisters and friction are the only remedies from which he had ever seen benefit result, and these acted merely as palliatives, even in the early and uncomplicated stage of the thymous tumor. All other resources failing, Mr. Burns thought an attempt to remove the diseased gland, although a most perilous operation, warrantable. He had twice effected it on the *dead* subject, in a manner which will be best described by himself.

"I made an incision on the front of the neck just above the

---

* The laryngeal artery, a branch of the superior thyroid, and the laryngeal nerve, coming from the eighth pair, enter the cavity of the larynx between the hyoid bone and thyroid cartilage.

sternum," (the part where the swelling of the thymus will be felt, when so much enlarged as to produce alarming symptoms, and where it is covered by the fascia and sterno-hyoid and thyroid muscles) " and between the sterno-hyoid muscles, as in the operation of tracheotomy. By this cut, the rounded knob of the diseased thymus was exposed. Having done this, I next insinuated the fore-finger between the gland and the adjacent parts till the former was insulated as far as I could reach. After this, by a pair of polypi forceps cautiously introduced between the mediastinum and the gland, I grasped the tumor and wrenched it from its connections."

Injury of the large vessels might be avoided by proper caution: hæmorrhage from the arteries of the gland would be prevented by the mode of operating; or, if arising, might be controlled by the sponge: and consequent inflammation could scarcely be dreaded in the state of debility which the disease must have necessarily induced. Such are the arguments, probably more plausible than solid, employed by Mr. Burns, in speaking of an operation; which, we think, he would have had occasion to mention in very different terms, if ever it had been his lot to attempt it in the *living* subject. The very idea of tearing out a morbid, and perhaps preternaturally adherent, mass from between the plates of the delicate pleuritic membrane, is enough to appal the most hardy operator. But this, formidable as it is, constitutes not the sole objection to the procedure. Thymous enlargement is commonly of constitutional origin; and often complicated, not only with mesenteric induration, as expressly noted by Mr. Burns but with scrofulous lesion of other organs, particularly the bronchial glands. And it will generally be found that, ere the diseased thymus has acquired such a volume as to produce baneful effects by its mechanical pressure, other important parts of the system have been so deeply contaminated, as to destroy the prospect of permanent benefit, even if mediastinal tumor could be successfully extirpated. Again, with respect to the obstruction of the mesentery, frequently existing in these cases, we differ from Mr. Burns, who believes it to be dependent upon the pressure of the enlarged thymus on the subclavian vein. To us it appears that the thoracic and abdominal affections should be considered, not in the light of cause and consequence, but as common, and frequently simultaneous, in their origin from the same morbific source.

*Pathology of the thyroid gland.* This organ is subject to several forms of morbid affection. *Inflammation* of it sometimes terminates in suppuration, which may be general or partial. The abscess is flat on the surface; tense; and, by its pressure, productive more or less of uneasiness, according to the portion of the gland in which it has formed. Swelling of the right lobe is least mischievous; that of the cross slip induces principally dyspnœa from its situation, just over the trachea. Dysphagia constitutes the more urgent feature where the left lobe is affected, and respiration is also disordered. But, while these circumstances decide the *character* of the symptoms, their *intensity* is regulated by the condition of the fascia and muscles. In chronic affections of the gland, as bronchocele, dropsy, and scrofulous enlargement, these coverings yield before the tumor: in inflammation and scirrhus they resist its increase. Hence, dysphagia and dyspnœa are proportionately more urgent in the latter class, than in the former. Thus may be explained the occurrence of difficult respiration and deglutition in some cases of partial or trifling enlargement of the gland; while in others, where the organ is universally tumefied, these phænomena exist only in a slight degree. If the inflammation of this gland should defeat all the attempts at resolution, and suppuration ensue, the pus ought to be discharged by puncture as soon as its existence is ascertained; for not only has it been known to accumulate, from delay, to the amount of some pounds, but spontaneous effusion into the gullet or wind-pipe may be dreaded. The wound will soon heal if the inflammation have been simple, but this event may be retarded by certain peculiarities of constitution. Induration and enlargement of the gland sometimes continue, in scrofulous subjects, after the contents of the cysts have been discharged. Against these, the treatment indicated in analogous cases, must be employed. *Chronic enlargement* sometimes succeeds parturition, without apparent cause. The increase is slow; and, until far advanced, not productive of uneasiness. Such tumors sometimes spontaneously disappear, sometimes suppurate. They require no peculiar treatment. *Dropsy* of the gland may arise from effusion of fluid into the cells composing it. Fluctuation, and transparency of the tumor when examined by candle-light, characterize this state. But if the fluid be tinged with blood, diagnosis will be rendered obscure. The cells are sometimes filled with blood itself. The cure may be accomplished by incision of the cyst, or by puncture and injection of diluted wine as in hydrocele. Stimulating applications to the exterior will sometimes produce absorption of the fluid. In a case of dropsy, complicated with bronchocele, a seton removed the fluid, but made no impression on the glandular enlargement.

*Bronchocele* is an indolent enlargement of this gland, often occurring, rarely suppurating, of long continuance, and productive of trifling inconvenience, proportionately to its size. The structure of the organ is little altered, except that its cells are enlarged, and contain a glairy fluid coagulable by alcohol. It commonly arises between the eighth and twelfth year; increases slowly for a time; afterwards more rapidly; is, at first, nearly painless, mobile, compressible; in its later stages, solid and adherent. Its effects on respiration will be determined by the locality of the swelling and state of the muscles. The lateral lobes of the gland only implicated, a hollow is formed on the front of the throat during each act of deglutition; but if the enlargement affect also the cross slip, this depression is never distinctly seen. When one lateral lobe is exclusively swollen in this or any other species of thyroid enlargement, the tumor is usually seated just upon the common carotid artery; and, receiving an impulse from that vessel, may resemble aneurism: but the diagnosis is easy. Mr. Burns met with a case, in which the left lobe of the enlarged gland, beneath the sterno-mastoid muscle, was "lobulated and clustered into small processes, precisely resembling a chain of enlarged concatenated glands." This curious structure extended backward into the space between the sterno-mastoid and trapezius muscles. It is of consequence that this morbid phænomenon should be correctly known; as the belief of the existence of diseased absorbent glands, which it might inspire, would at once deter the surgeon from extirpating the thyroid tumor, although in every other respect favourably circumstanced for an operation. Such an operation is practicable, but it must be performed early, ere the tumor has become adherent to the capsule of fascia in which it is enclosed, and deeply connected with the surrounding parts. If, however, from any such cause, removal of the gland be contra-indicated, the superior thyroid arteries may be tied. The enlarged size of these arteries, and their superficial situation, render such a procedure perfectly easy.* Thus the increase of the

---

* The arteries of the thyroid gland are commonly four in number: the two superior, furnished by the external carotid; the two inferior, by the swelling will be retarded, and its volume sometimes so much reduced as to admit of extirpation, when previously, from this cause alone, such an attempt would have been unwarrantable. Bronchocele in young females often spontaneously disappears on the establishment of the menstrual flux. With respect to the treatment, Mr. Burns strongly recommended topical bleeding; regular and long-continued friction with a flannel on which hair-powder has been sprinkled, for twenty minutes at a time, thrice a day; friction with camphorated mercurial ointment; blisters; electricity. He had never witnessed benefit from the employment of burnt sponge or other constitutional remedies, except such as regulate the functions of the intestinal canal.

*Carcinoma* may affect the thyroid gland: which, in this disease, enlarges slowly, sometimes without evident cause; sometimes after the infliction of external violence. The swelling has an irregular surface, and stony consistence; the pain is lancinating, and strikes principally upward; breathing and deglutition are both affected, but unequally, according to the situation of the diseased portion of the gland. The muscles are rigid and adherent to the tumor; the integuments, in an advanced stage, matted and puckered. Cysts, containing a sanious fluid, arise sometimes near the surface; they enlarge, and by their pressure induce dreadful distress of respiration and swallowing. At length, when the patient is sinking, one of the most prominent collections gives way, and the discharge is succeeded by temporary relief; and, in the same manner, recurrence of the symptoms is successively mitigated, till they become so urgent as no longer to admit of even this palliation. A most interesting case and dissection of cancerous thyroid gland is here recorded. In addition to the morbid appearances of the organ itself, there were found thickening and induration of the *neurilema* of the eighth and sympathetic nerves; and the right "internal jugular vein, from a little below the angle of the jaw down to near the chest, was completely obliterated. The blood was sent across to the opposite vein by a large communicating vessel which ran parallel to the body of the hyoid bone."

---

subclavian, from a trunk common to other vessels. Sometimes, an anomalous branch is given to the gland by the arteria innominata; and enters the cross slip, just in front of the trachea: it occasionally supplies the absence of one of the regular arteries.

The terrible affection, denominated by Mr. Abernethy *medullary sarcoma,* may attack the substance of the gland. The swelling is, at first, uniform and elastic; but gradually assumes an uneven surface. The muscles are rigid; the integuments tense, and, in an advanced case, dark-coloured; the veins, on the most prominent parts, varicose. The countenance is squalid; nights restless; respiration difficult and wheezing; deglutition much obstructed: pulse frequent; pain severe, lancinating, almost constant, though prone to exacerbations. The patient is commonly, not always, destroyed, ere the tumour has become large. By many surgeons this malady is confounded with fungus hæmatodes. Mr. Burns, however, has shown them to differ essentially in their internal characters. The mass which fills the tumor, in medullary sarcoma, is of uniform pulpy consistence, resembling in hue the cortical substance of the brain, and it may be completely washed out from its capsule, with the exception of "a number of flocculi hanging from its inner surface;" while the "brain-looking matter" of fungus hæmatodes will be equally separated by the same treatment; but the numerous bands, by which the body of the tumor is intersected, will remain.

We have now to trace the parallel between cancer and fungus hæmatodes, which Mr. Burns has presented in a diffuse but not very clear and distinct way. Here, as on all other occasions, we shall pursue our own method of analysis, and contrast carcinoma with the fungous tumor, under the three following aspects: Their phænomena *previously to* ulceration; their characters and progress *after* that period; and their peculiarities of morbid structure. —1. *Carcinoma.* It were useless to retrace the characters which we have just delineated as marking this disease. Incompressibility, stony hardness, and an irregular surface, are among the most conspicuous. — *Fungus hæmatodes.* Tumor, at first, small, colourless, elastic, firm in proportion to the depth of its site; tense, if covered by a fascia; surface irregularly projecting, and the prominent points soft, not hard as in cancer, even yielding a delusive sense of fluctuation, and "generally marked with small varicose veins from which they derive a bluish livid colour." Beneath these, are seated the rudiments of the future fungi. — 2. *Carcinoma.* Margins of the ulceration thin, livid, glassy, often retroflected; fungus sometimes arises, but sloughs like the substance of the tumor; and thus a deep cavern, with livid, undetermined, ragged, and occasionally reverted edges is formed.—*Fungus hæmatodes*. Bloody ichorous discharge in small quantity when the skin over a protuberance bursts; fungus rapidly springing from the orifice, and soon folded over its margin; often bleeding profusely, and constantly "smeared over with a film of bloody lymph;" sometimes destroyed in consequence of its neck being tightly begirt by the skin; loss of substance never occurring but in this way. The lymphatic glands become at length contaminated, and the progress of the secondary affection is more rapid than the original. — 3. *Carcinoma* is internally composed of two clearly dissimilar substances: the one hard and fibrous; the other softer and seemingly unorganized. The first predominates, and forms septa paler than the soft part, opaque, unequal in length, breadth, and thickness, variously disposed so as to form a solid mass, or several irregular cavities containing the inorganic portion; which may be semi-transparent, bluish, of the consistence of softened glue; or oleaginous and cream-like in hue and fluidity. But the proportion and distribution of these substances are infinitely various. Most commonly, the fibrous part is predominant and condensed into a nucleus, from which the septa go off, like radii, in every direction. Sometimes there is an irregularly shaped, uniformly hard mass, without defined structure. Sometimes the fibrous portion forms cells from which the pulpy substance can be readily pressed; or there are cysts of various sizes, containing a bloody or dark chocolate-like fluid, and occasionally lodging a fungous tumor; or, lastly, parts of the morbid mass may become changed into a cartilaginous substance, wherein bone is now and then deposited.—*Fungus hæmatodes* consists of a soft pulpy matter, miscible with water, and hardened by acids and boiling; in hue and consistence resembling medullary substance: the colour, when the tumor is small, pale grey or brownish red; but, when large, the different portions, separated by capsules, differ in appearance, resembling brain, boiled yolk of egg, coagulated blood, liver, or assuming a deep yellow colour. Sometimes there are small excavations lodging a bloody fluid. The different lobes, composing the mass, are always separated by thin membranous septa, and sometimes hard, cartilaginous, or even ossified. In colour, the fungi are darker than the rest of the mass. The neighbouring bones, after a time, become soft; their cancelli are destroyed, and their situation is occupied by a soft mass of cineritious matter. The fibrous texture of the muscles, but not their shape, disappears; and they assume "a dusky white or brown colour." No instance of fungus hæmatodes, affecting the thyroid gland, is cited by Mr. Burns. He had seen one part of a diseased breast invaded by it, the other by cancer, at the same time; and he had reason to "believe" that scrofula and fungus hæmatodes may co-exist in the same tumor.

The situation of the carotid artery, with respect to the tumor, will vary according to the nature of the morbid affection. In carcinoma, the swelling is firm, and pushes aside that vessel, the internal jugular vein, and eighth nerve. In medullary sarcoma and fungus hæmatodes, the morbid parts are soft, and the carotid artery is frequently imbedded in their substance.

When the structure of the thyroid gland is attacked by either of these malignant affections, external remedies will avail nothing; nor will ligature of its arteries repress the growth of the tumor, or destroy its specific character. The whole must be extirpated, while it is yet small and unadhering. It is a dangerous operation, but has repeatedly been performed with success. The surgeon should begin by making an eliptical incision over the tumor, if it be large or the integuments diseased, in a perpendicular direction. The integuments, on each side, are then to be dissected back; and the four thyroid arteries, which will be reached by the finger insinuated between the skin and muscles, successively secured. This done, the vessels are to be cut through between the ligature and gland; the sterno-hyoid and thyroid muscles to be divided above and below; and the tumor detached from the trachea and gullet "by cautious working with the fingers." The swelling, when large, has been known to descend to the margin of the sternum, and adhere to the arteria innominata.

*Œsophagotomy.* From the structure of the pharynx and œsophagus, a substance which has passed the tapering part of the former, may be detained in the commencement of the latter. In this situation, it can neither be forced down by the probang nor withdrawn, and may require to be extracted by an operation; the dangers and difficulties of which have been over-rated by viewing the relations of the parts in their natural state. The foreign substance, impacted in the gullet, affords a sure guide to the knife of the surgeon. The carotid artery is pushed aside out of danger. The twigs of the recurrent nerve, and thyroid branch of the inferior thyroid artery, only are exposed; and these, if the finger be employed to denude the œsophagus before it is opened, will be safe. Mr. Burns once saw an anomalous artery, arising from the arteria innominata, and traversing this part of the gullet in its course up the neck. The possibility of irregular distribution of the arteries should always be borne in mind by the operator.

(To be continued.)

---

### *Mr. Hill's Essay on the Nature and Cure of Insanity.*

